# Impact of obesity on taste receptor expression in extra-oral tissues: emphasis on hypothalamus and brainstem

**DOI:** 10.1038/srep29094

**Published:** 2016-07-08

**Authors:** D. Herrera Moro Chao, C. Argmann, M. Van Eijk, R. G. Boot, R. Ottenhoff, C. Van Roomen, E. Foppen, J. E. Siljee, U. A. Unmehopa, A. Kalsbeek, J. M. F. G. Aerts

**Affiliations:** 1 Department of Medical Biochemistry, Academic Medical Center, Amsterdam, 1105 AZ, The Netherlands; 2 Department of Endocrinology and Metabolism, Academic Medical Center, Amsterdam, 1105 AZ, The Netherlands; 3 Department of Genetics and Genomic Sciences, Icahn Institute for Genomics and Multiscale Biology, Icahn School of Medicine at Mount Sinai, New York, NY 10029, USA; 4 Department of Biochemistry, Leiden Institute of Chemistry, Leiden, 2333 CC, The Netherlands; 5 Hypothalamic Integration Mechanisms, Netherlands Institute for Neuroscience, Amsterdam, 1105 BA, The Netherlands

## Abstract

Sweet perception promotes food intake, whereas that of bitterness is inhibitory. Surprisingly, the expression of sweet G protein-coupled taste receptor (GPCTR) subunits (T1R2 and T1R3) and bitter GPCTRs (T2R116, T2R118, T2R138 and T2R104), as well as the α-subunits of the associated signalling complex (αGustducin, Gα14 and αTransducin), in oral and extra-oral tissues from lean and obese mice, remains poorly characterized. We focused on the impact of obesity on taste receptor expression in brain areas involved in energy homeostasis, namely the hypothalamus and brainstem. We demonstrate that many of the GPCTRs and α-subunits are co-expressed in these tissues and that obesity decreases expression of T1R3, T2R116, Gα14, αTrans and TRPM5. *In vitro* high levels of glucose caused a prominent down-regulation of T1R2 and Gα14 expression in cultured hypothalamic neuronal cells, leptin caused a transient down-regulation of T1R2 and T1R3 expression. Intriguingly, expression differences were also observed in other extra-oral tissues of lean and obese mice, most strikingly in the duodenum where obesity reduced the expression of most bitter and sweet receptors. In conclusion, obesity influences components of sweet and bitter taste sensing in the duodenum as well as regions of the mouse brain involved in energy homeostasis, including hypothalamus and brainstem.

Taste perception is an important aspect in the control of food intake. Taste is mainly sensed by taste receptor containing cells located in the taste buds distributed in the different gustatory epitheliums in the tongue, palate, larynx and epiglottis. The sensing of sweet, umami and bitter taste is mediated by two G protein-coupled taste receptor (GPCTR) families: the T1R family, which is mainly involved in the sensing of sweet and umami taste-like signalling molecules and the T2R family, involved in the sensing of bitter taste-like signalling molecules^1^. The T1R family consists of three different GPCTRs that generate at least two heterodimeric receptors: T1R1+T1R3 associated with umami taste sensing and T1R2+T1R3 associated with sweet taste sensing^1^,^2^. In mice the T2R family consists of at least 36 distinct taste receptor members, which individually sense bitter taste like molecules^3^. The human T2R16 selectively recognizes β-glucopyranosides^4^, while the human T2R38 recognizes phenylthiocarbamide (PTC)^5^. The functional importance of the latter two human receptors was demonstrated by the finding that overexpression of either receptor in mice increases food avoidance^6^.

Although the T1R and T2R receptor families drive different taste perceptions, they share similar downstream G protein-coupled signalling pathways. In particular, the taste specific α-subunit of the G protein α-gustducin (αGust) is coupled to both receptor families and has been described as critical for sweet and bitter taste responses^7^. Nevertheless, αGust knockout animals still preserve a moderate sensitivity to some bitter compounds and to sweet compounds in higher mM concentrations^7^,^8^, suggesting that other G protein coupled α-subunits are also involved in the transduction of taste signal responses. Interesting candidates in this context are α-transducin (αTrans) and α14 (Gα14) transducing sweet, umami and bitter taste signalling^9^,^10^,^11^,^12^,^13^. Activation of the taste GPCTRs triggers the release of G protein β-γ subunits stimulating phospholipase C β2 (PLCβ2) and second messengers increasing cytosolic Ca^2+^. In secretory cells the increase in cytosolic Ca^2+^ next activates the transient receptor potential cation channel M5 (TRPM5), which induces membrane depolarization and action potential generation^1^,^14^.

The initial tasting of food is mediated by GPCTRs located in gustatory tissues such as the anterior and posterior tongue and palate^15^, rallying the signal via afferent nerves to the rostral part of the nucleus of the solitary tract (NTS) in the brainstem. From there the information is transferred to the parabrachial nucleus (PBN) and by an alternative pathway to the thalamus. Next, the PBN projects to the hypothalamus and amygdala to regulate feeding and taste memory formation. The projections to the thalamus end at the gustatory cortex where taste perception is integrated^16^.

T1R and T2R family members have also been found in chemosensory cells located in extra-oral tissues like upper airways^17^,^18^, testis^19^, gastrointestinal (GI) tract^20^,^21^,^22^ and brain areas related to energy homeostasis (hypothalamus and brainstem)^23^,^24^. In particular, their presence in chemosensory cells in the GI tract might not be related to a sensation of taste, but to the modulation of food intake, gastric distension and gastric emptying through the secretion of gut hormones^25^,^26^,^27^. The fact that sweet and bitter receptors can modulate endocrine functions and are present in extra-oral tissues, suggests that they may play a role in the control of energy homeostasis and furthermore in the development of obesity and diabetes. Few reports exist on the regulation of taste receptors in extra-oral tissues in obese and insulin resistance settings^28^. We investigated whether the nutritional status influences the expression of taste receptors and known downstream genes in oral and extra-oral organs in mice. Our interest focussed on taste receptor expression in the brain and in particular in the brainstem and hypothalamus, in view of their importance in the control of energy homeostasis.

## Material and Methods

### Animals and experimental design

Eight to ten week old C57Bl/6 and ob/ob mice (purchased from Envigo, Venray, The Netherlands) were kept in group housing conditions in a room with constant temperature (23 ± 2 °C) and a 12h/12h light/dark cycle (lights on at 07:00). Lean C57BL/6 mice (wt *ad libitum*) (n = 6) and obese ob/ob mice (n = 6) on a C57BL/6 background (ob/ob) had access to chow food *ad libitum*. Another group of mice (diet induced obese; DIO) (n = 6) had access to a 20% kcal protein, 20% carbohydrates and 60% fat diet (HFD) *ad libitum* for sixteen weeks (Research Diets D12492, NJ, USA). In addition, an overnight 16 hour fasting (lean fasting) group of C57BL/6 mice (n = 5) was included. Food intake and body weight was assessed once a week in all groups. All animal experiments were performed according to regulations in The Netherlands and were approved by the institutional ethics board of the Royal Academy of Sciences (KNAW) (DEC protocol number: 101943). All animals were sacrificed at 11:00 am; they were put asleep in a CO_2_ chamber and then decapitated prior to the removal of organs. Gustatory tissue (tongue) and extra-oral tissues (trachea, lungs, duodenum, liver and brain) were dissected and immediately snap frozen in liquid nitrogen. The frozen brains were dissected in four different areas: hypothalamus (HT), brainstem (BT), hippocampus (HP) and cingular cortex (Ctx).

### OGTT, plasma Insulin and plasma leptin assessment

An oral glucose tolerance test (OGTT) was performed after four hours fasting in the lean C57BL/6 *ad libitum* (n = 6) and the DIO group (n = 6) 10 weeks after the beginning of the HFD exposure. A basal blood sample was taken by a tail cut, followed by a 20% D-glucose oral gavage bolus (1 g/kg, D-glucose was obtained from Sigma, St Louis, USA). Blood samples were taken at 5, 10, 15, 30, 60 and 120 minutes after the glucose bolus. The area under the curve of plasma glucose levels was calculated for each animal, DIO animals with a statistical significant higher area under the curve compared to the lean *ad libitum* animals were considered for further gene expression analysis. Plasma glucose, insulin (insulin ELISA kit, Crystal Chem, IL, USA) and leptin (leptin ELISA kit, R&D Systems, MN, USA) were also measured. The HOMA index was calculated using the following formula: (mM glucose value^*^μunits/ml insulin value)/22.5.

### DIO and ob/ob mice characterization

DIO (p = 0.001) and ob/ob (p = 0.001) mice had a significantly higher body weight compared to the *ad libitum* controls, accompanied by higher plasma glucose (DIO (p = 0.001) and ob/ob (p = 0.001) mice) and plasma insulin (DIO (p = 0.024) and ob/ob (p = 0.001) mice levels. Leptin levels were only detectable in the DIO and *ad libitum* groups and differed significantly (p = 0.006) (Supplemental Table S1). OGTT in DIO mice (p = 0.001) was significantly different from the OGTT of *ad libitum* controls. Additionally, DIO (p = 0.001) and ob/ob mice (p = 0.001) presented a higher HOMA-IR compared to the *ad libitum* control animals, indicating insulin resistance (Supplemental Table S1).

### Neuronal cell culture experiments

The adult mouse hypothalamic cell line (mHypoA-2/12p; CELLutions, University of Toronto Innovations Foundation, Canada) was cultured in DMEM high glucose (Life Technologies, Waltham, MA USA) with 10% fetal bovine serum and 1% penicillin/streptomycin and maintained at 37 degrees under 5% CO_2_ and grown until 70% confluence. The following day, the cells were washed in phosphate buffered saline (PBS) and cultured in DMEM high glucose, supplemented with recombinant human Leptin (0, 50 and 100 nM) (R&D systems, Minneapolis, USA) either for 1 hour (n = 6 for each concentration) or for 7 hours (n = 6 for each concentration). To study the effect of glucose, the cells were cultured in DMEM without glucose + 0.5 mM glucose, after that they were washed in PBS and then DMEM without glucose + 5 mM glucose (n = 8) or DMEM without glucose + 0.5 mM glucose was added (n = 8) (D-glucose was obtained from Sigma, St Louis, USA) for 1 hour (n = 4 for each concentration) or for 7 hours (n = 4 for each concentration). After stimulation, mRNA was extracted. Since we compared gene expression of treated cells to untreated cells at one time point, we used statistical analysis by two-way ANOVA. We related values of expression of specific genes to the mean of all expression values of that specific gene for cells exposed to 0.05 mM glucose and in the case of leptin to mean of the values of the cells exposed to 0 nM leptin. After this normalization, a mean and standard error was determined.

### RNA isolation and RT PCR

Tongue, trachea, lungs, duodenum, liver and brain areas were lysed in Trizol Reagent (Life technologies, Carlsbad, California, USA), RNAse free chloroform was added and the transparent RNA phase was taken in a new RNAse free tube where 70% alcohol was added. After this, RNA was further purified using the Nucleospin II extraction kit (Macherey-Nagel GmbH, Duren, Germany). In the case of the hypothalamic neuronal cell experiments, the Nucleospin II extraction kit (Macherey-Nagel GmbH, Duren, Germany) was used to extract RNA. cDNA was synthesized according to the Invitrogen cDNA synthesis kit. Gene expression analysis was performed using a Bio Rad MyIQ Real Time PCR detection system. The expression levels were normalized to the mean of P0 and GAPDH expression levels. The primers used for each gene investigated are presented in Supplemental Table S2.

### LNA *in Situ* Hybridization and immunostaining

The C57BL/6 mice (n = 4) used for the *in situ* hybridization and immunostainings were first transcardially perfused, with 250 ml of 0.9% RNAse free saline solution and 250 ml of paraformaldehyde diluted in phosphate buffer (0.1 M, pH 7.2). Brains were removed afterwards and post-fixed overnight in 4% paraformaldehyde at 4 °C. After this period the brains were kept in cryo-protectant in 30% RNAse free sucrose solution until the brains sank. Immediately after that, the brains were cut in 20 μm coronal brain slices and frozen at −80 °C in RNAse free cryo-protectant. Prior to the *in situ* hybridization, free floating sections were rinsed in RNAse free PBS for five minutes, followed by a PBS- 0.05% Triton X-100 incubation for 10 minutes. After this, the sections were rinsed in PBS for 5 minutes and rinsed in 0.2 M HCl solution for 10 minutes. The sections were rinsed again in PBS for 5 minutes. Ninety minutes prehybridization in a humidified chamber at 60 °C was performed in a hybridization mixture (hybmix) with final concentrations of 50% (v/v) deionized formamide, 600 mM NaCl, 10 mM HEPES, 50× Denhardt’s, 1 mM EDTA, and 10 mg/ml denatured fish sperm DNA (Life Technologies, Waltham, MA USA). 5′-FAM labeled T2R116 and Gα14 LNA-2′ OmethylRNAprobes were diluted in Hybmix to a final concentration of 5 nM, denatured at 95 °C for 5 minutes and cooled on ice. Sections were hybridized overnight at 60 °C and subsequently washed for 5 min each in 2× SSC, 0.5× SSC, and 0.2× SSC at 60 °C and for 5 min in Tris buffered saline (TBS) at room temperature (RT). Next, sections were incubated in anti-FAM-Alkaline Phosphatase (Roche, Mannheim, Germany) 1:3000 in (0.25% (w/v) gelatin and 0.5% (v/v) Triton X-100 in TBS, pH 7.6, for 3 h at RT. Slides were washed 5 min in buffer 1 (100 mM Tris, 150 mM NaCl at pH 7.5) twice, and after a prewash in buffer 2 (100 mM Tris–HCl, pH 9.5, 100 mM NaCl, and 5 mM MgCl2), the color was developed using NBT-BCIP solution (337.5 mg/ml NitroBlue Tetrazolium Chloride (Sigma, St Louis, USA)), 175.4 mg/ml 5-bromo-4-chloro-3-indolyl phosphate toluidine salt (Roche, Mannheim, Germany), 240 mg/ml levamisole in 100 mM Tris–HCl, pH 9.5, 100 mM NaCl, and 5 mM MgCl_2_ for 3 h under dark conditions. Sections were then washed in distilled water, incubated in methanol for 5 min, rinsed again twice in distilled water and once again in TBS. Some sections hybridized with the T2R116 were further incubated overnight with a rabbit anti-Gα14 primary antibody (1:1000, abcam, Cambridge, UK), some others in rabbit anti-αGust primary antibody (1:1000, Santa Cruz, Dallas, USA) and some others were incubated overnight in rabbit anti-PLCβ2 primary antibody (1:1000, abcam, Cambridge, UK). After the primary incubation the sections were rinsed with PBS 0.01 M and incubated with donkey anti rabbit secondary antibody (1:400, Vector technologies, CA, USA) for 1 hour and again rinsed for 10 min. Next avidin-biotin complex incubation was performed (1:500, Vector Laboratories, CA, USA) for an hour and then rinsed 3 times with PBS for 10 min. Finally tissues were reacted with diaminobenzidine (10 mg/100 ml TBS) and hydrogen peroxide (10 μl, 30% H_2_O_2_) for 6 min and rinsed again with PBS for 10 min. Finally, the sections were coverslipped with Aquamount (Merck) and stored at 4 °C.

Co-localization of the *in situ* hybridization signal and antibody staining was analyzed by spectral imaging analysis using a CRi Nuance FX camera and software, Image pro (Media Cybernetics, Silver Spring, MD, USA), in combination with software developed at the Netherlands Institute for Neuroscience. Individual spectra of NBT-BCIP and DAB were made by collecting light in cubes of 20nm along a 200–600 nm spectrum. The double stained slides were then analyzed with the spectral library and unmixed into individual black and white images, representing the respective signal of each reaction products. Fluorescent pseudo-colors were then applied to each signal to enhance color separation and further visualize co-localization. The pseudo-color co-localization analysis was performed as previously described^29^.

### Cluster analysis and statistical analysis

Unsupervised hierarchical clustering was performed using complete linkage and Spearman rank correlation distance on the 11 normalized taste receptor genes using software implemented in Genepattern (Broad Institute, MIT and Harvard, USA). The *z*-scores were calculated within tissue type in the following manner: mean of expression of taste receptor gene in each nutritional group – average of expression of taste receptor gene within a specific tissue type/STDEV. Color in the heat maps reflects the relative gene expression level; with red being higher expressed and blue lower expressed than the mean taste receptor expression within tissue value.

The RT-PCR mRNA expression data of the different taste receptors during the different experimental conditions are presented as mean ± SEM. Two-way ANOVA and one-way ANOVA were performed using SPSS statistical package version 19. Pairwise comparisons were evaluated with a LSD post-hoc test. Significant values were set at p < 0.05.

## Results

### Expression of sweet GPCTR subunits and bitter GPCTR receptors and associated a-subunits in the brain of lean mice

**Figure 1:**
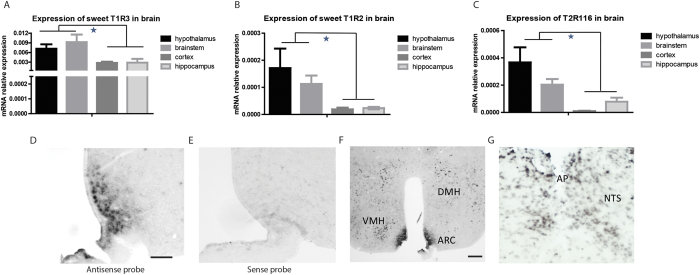
Sweet and bitter receptors are expressed in the brain of lean wild-type C57Bl6 mice. (**A**) Sweet receptor subunit T1R3 mRNA expression in brain areas as assessed by real time PCR. T1R3 is higher expressed in the hypothalamus and brainstem. (**B**) Sweet receptor subunit T1R2 mRNA expression in brain areas. Again, the hypothalamus and brainstem present the highest expression in brain. (**C**) Bitter receptor T2R116 mRNA expression in brain areas. The hypothalamus and brainstem present the highest expression in brain. (**D**) ARC T2R116 mRNA expression after using anti sense hybridization probe. Positive signal could only be observed after anti sense probe hybridization. The scale bar represents 0.5 mm. (**E**) ARC T2R116 mRNA expression after using sense hybridization probe. Almost no signal could be observed after sense probe hybridization. (**F**) T2R116 mRNA expression in the hypothalamus. High expression levels were found in the ARC, VMH and DMH. The scale bar represents 0.5 mm. (**G**) T2R116 mRNA expression in the brainstem. High expression levels were found in the NTS. Expression of mRNA was normalized to that of P0 and GAPDH.

We analyzed the pattern of expression of sweet taste receptors subunits (T1R2 and T1R3) and bitter taste receptors (T2R116, T2R118, T2R138 and T2R104) in lean C57BL/6 mice throughout different brain areas including HT, BT, HP and Ctx. One-way ANOVA showed no significant differences between the geometric mean expression of the reference genes (P0 and GAPDH) in the four brain areas (p = 0.921). Of all the taste receptor genes analyzed, T1R3, T1R2 and T2R116 showed the highest relative expression throughout the different brain areas. Statistical analysis by ANOVA revealed significant differences in T1R3 expression among the brain areas evaluated (p = 0.001) (Fig. 1A). The HT shows higher T1R3 mRNA compared to the Ctx (p = 0.030). The BT as well presented significantly higher T1R3 mRNA compared to the HP (p = 0.001) and Ctx (p = 0.001). The same pattern was observed for T1R2 mRNA expression (p = 0.001) (Fig. 1B), wherein the T1R2 mRNA in the HT and BT was higher compared to that in the HP (p = 0.001) and Ctx (p = 0.001). Of the analyzed bitter taste receptors, T2R116 mRNA expression was significantly higher in HT compared to HP (p = 0.013) and Ctx (p = 0.007). Also the BT shows higher T2R116 mRNA expression compared to HP (p = 0.001) and Ctx (p = 0.001) (Fig. 1C).

To explore which hypothalamic and brainstem nuclei express the T1R3, T1R2 and T2R116 receptors, LNA *in situ* hybridizations in C57BL/6 lean mice brains were performed. The specificity of the *in situ* hybridization procedure was confirmed by comparison of anti-sense and sense LNA probes hybridization. There was a positive signal with antisense probe hybridization (Fig. 1D), while almost no signal was detected with sense probe hybridization (Fig. 1E). Previously it has been demonstrated that T1R2+T1R3 mRNA was expressed in the hippocampus, arcuate nucleus (ARC) and paraventricular nucleus of the hypothalamus^24^. T1R3 and T1R2 mRNA was detectable in the hippocampus and hypothalamus, with highest levels in the ARC. Similar patterns were found for T2R116, showing mRNA in the CA fields and dentate gyrus of the HP, in the insular cortex (data not shown), and within the HT in the ARC (Fig. 1D,F), the ventromedial nucleus (VMH) (Fig. 1F) and the dorsal part of the dorsomedial nucleus (DMH) (Fig. 1F). In addition, T2R116 mRNA was detected in the rostral and caudal part of the NTS in the BT (Fig. 1G).

**Figure 2:**
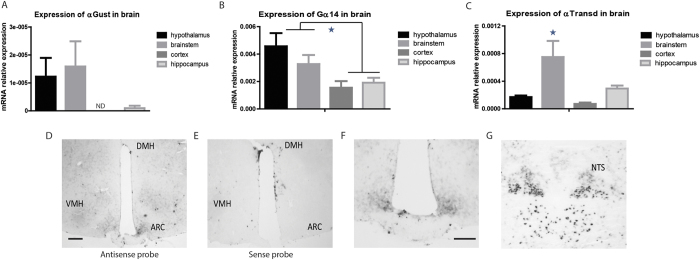
GPCTR associated α-subunits are expressed in key homeostatic areas of the hypothalamus and brainstem of lean wild-type C57Bl6 mice. (**A**) GPCTRs α subunit Gustducin (αGust) mRNA expression in brain areas. α Gustducin expression in the brain was very limited. ND = not detected. (**B**) GPCTRs α subunit Gα14 mRNA expression in brain areas. The hypothalamus and brainstem present the highest expression in brain. (**C**) GPCTRs α subunit Transducin (αTransd) mRNA expression in brain areas. α Transducin is mainly expressed in the brainstem. (**D**) Gα14 mRNA expression in the hypothalamus after using anti sense hybridization probe. The scale bar represents 0.5 mm. (**E**) Gα14 mRNA expression in the hypothalamus after using sense hybridization probe. (**F**) Gα14 mRNA expression in the ARC. The scale bar represents 0.5 mm. (**G**) Gα14 mRNA expression in the NTS.

Next we analyzed the mRNA expression of different α-subunits of GPCTRs signalling. αGust mRNA (Fig. 2A) and αTrans mRNA (Fig. 2C) were both poorly expressed in all brain areas. An exception in this respect formed the higher expression of αTrans mRNA in the BT (Fig. 2C). Gα14 mRNA expression was found to be high in all brain areas (Fig. 2B); ANOVA indicated significant differences between brain areas (p = 0.001), with higher Gα14 mRNA expression in HT compared to Ctx (p = 0.006) and compared to HP (p = 0.030). Gα14 mRNA expression in the BT was also higher compared to Ctx (p = 0.018).

Due to the higher Gα14 mRNA levels in the brain, compared to the other α subunits analyzed, LNA *in situ* hybridization was performed in order to identify the specific hypothalamic and brainstem areas that present Gα14 mRNA expression. Specific LNA *in situ* hybridization for Ga14 mRNA with antisense probes revealed expression in the HP and HT, especially ARC, VMH and DMH (Fig. 2D,F) and in the NTS and hypoglossal nuclei of the BT (Fig. 2G).

### Colocalization of taste receptors and downstream signaling mediators

**Figure 3:**
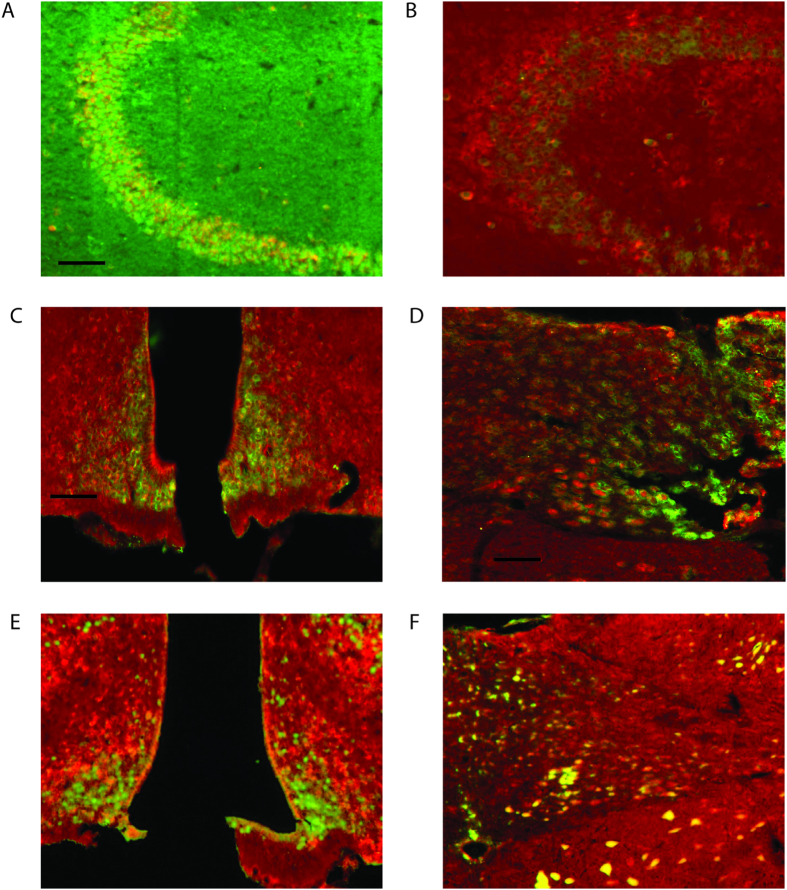
T2R116 positive cells present other taste receptor signalling pathway proteins. (**A**) T2R116 mRNA expression and α Gustducin (αGust) protein expression in the hippocampus. Clear overlap between T2R116 mRNA and α Gustducin protein expression. In green: T2R116, in red: αGust. The scale bar represents 0.5 mm. (**B**) T2R116 mRNA expression and Gα14 protein expression in the hippocampus. Almost no overlap between T2R116 mRNA and Gα14 protein expression. In green: T2R116, in red: Gα14. (**C**) T2R116 mRNA expression and Gα14 protein expression in the ARC. Colocalizations between the mRNA and protein signal. In green: T2R116, in red: Gα14. The scale bar represents 0.5 mm. (**D**) T2R116 mRNA expression and Gα14 protein expression in the NTS. Colocalizations between the mRNA and protein signal. In green: T2R116, in red: Gα14. The scale bar represents 0.5 mm. (**E**) T2R116 mRNA expression and PLCβ2 protein expression in the ARC. Colocalizations between the mRNA and protein signal. In green: T2R116, in red: PLCβ2. (**F**) T2R116 mRNA expression and PLCβ2 protein expression in the NTS. Colocalizations between the mRNA and protein signal. In green: T2R116, in red: PLCβ2.

In order to investigate whether the cells that express the different taste receptors, also contain the GPCTRs downstream signaling pathways, *in situ* hybridization for T1R2 and T2R116 was combined with immunohistochemistry for αGust, Gα14 and PLCβ2. Previously it was demonstrated that T1R2+T1R3 mRNA is expressed in the hippocampus, ARC and paraventricular nucleus of the hypothalamus^24^. Our combined spectrum analysis showed that T1R2 mRNA co-localized with αGust protein in the HP. T2R116 mRNA also co-localized with αGust protein in the HP, but hardly with Gα14 (Fig. 3A,B). In contrast, in the HT, specifically in the ARC, T2R116 mRNA co-localized with Gα14 protein (Fig. 3C); the same pattern was observed for the NTS, where T2R116 mRNA co-localized with Gα14 protein (Fig. 3D). Spectral imaging analysis indicated also co-localization of T2R116 mRNA in the ARC (Fig. 3E) and NTS (Fig. 3F) with PLCβ2 protein. It is known that the ARC, VMH, DMH and NTS are part of a circuit involved in the control of energy homeostasis^30^. The finding that T1R2 and T2R116 are expressed in these areas and that the cells that express the taste receptors also possess the associated downstream signaling pathways, led us to speculate that these taste receptors might contribute to the control of energy homeostasis. We therefore next examined taste receptors and downstream signaling mediators in obese animals.

### Obese mice show an overall decrease in GPCTR expression as compared to lean mice

**Figure 4:**
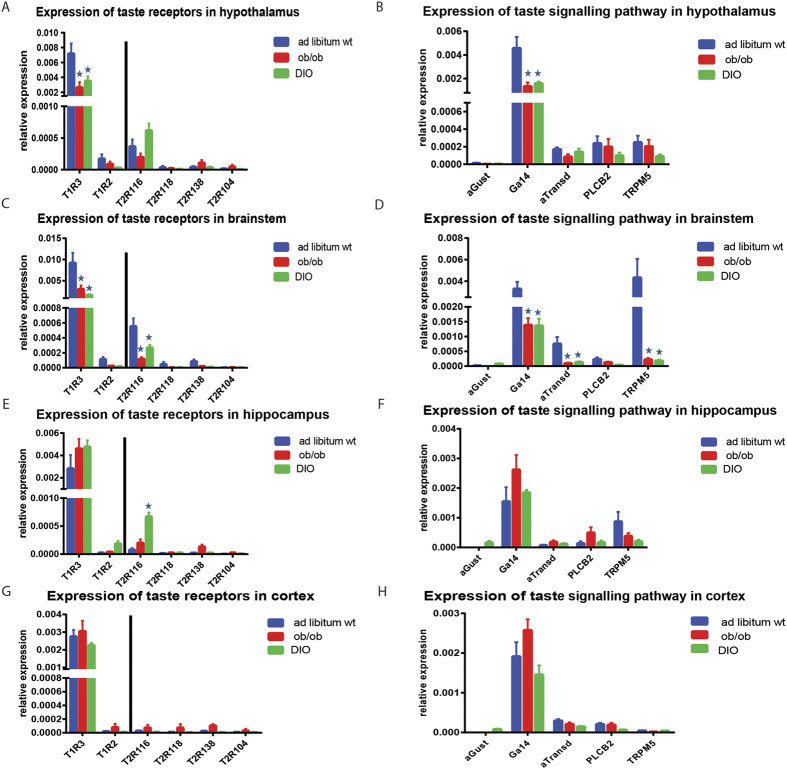
GPCTRs and GPCTR signalling pathway mRNA expression in brain is regulated by obesity. (**A**) Sweet and bitter taste receptor mRNA expression in hypothalamus of *ad libitum* wild type and obese (ob/ob and DIO) mice. T1R3 was reduced in ob/ob and DIO animals. (**B**) Downstream GPCTR signalling pathway mRNA expression in hypothalamus of *ad libitum* wild type and obese (ob/ob and DIO) mice. Gα14 was downregulated in obese (ob/ob and DIO) animals. (**C**) Sweet and bitter taste receptor mRNA expression in brainstem of *ad libitum* wild type and obese (ob/ob and DIO) mice. T1R3 and T2R116 were reduced in ob/ob and DIO animals. (**D**) Downstream GPCTR signalling pathway mRNA expression in brainstem of *ad libitum* wild type and obese (ob/ob and DIO) mice. Gα14, αTransd and TRPM5 were downregulated in obese (ob/ob and DIO) animals. (**E**) Sweet and bitter taste receptor mRNA expression in hippocampus of *ad libitum* wild type and obese (ob/ob and DIO) mice. T2R116 was upregulated in DIO animals. (**F**) Downstream GPCTR signalling pathway mRNA expression in hippocampus of *ad libitum* wild type and obese (ob/ob and DIO) mice. (**G**) Sweet and bitter taste receptor mRNA expression in Cingulate cortex of *ad libitum* wild type and obese (ob/ob and DIO) mice. There were no significant changes in expression. (**H**) Downstream GPCTR signalling pathway mRNA expression in Cingulate cortex of *ad libitum* wild type and obese (ob/ob and DIO) mice. There were no significant changes in expression.

To study the impact of metabolic status we investigated obese ob/ob and DIO C57BL/6 mice. We hypothesized that the HT and BT, major brain areas involved in the control of energy metabolism, might present significant changes in GPCTR and downstream signalling mediator gene expression, while the HP and Ctx would not be affected by the different nutritional states or only to a lesser extent. The expression of GPCTRs and related genes among brain areas of lean and obese mice was found to be different in several aspects. Obesity caused an overall downregulation of GPCTRs and related genes, specifically in the HT and BT (Supplemental Figure S1a). Statistical analysis by ANOVA indicated significant effects of nutritional status on the expression of GPCTR related genes in the HT (p = 0.001) and BT (p = 0.001). Multiple comparison post hoc tests of the HT (Table 1) indicated that mainly T1R3 and Gα14 were modulated during obesity (Fig. 4A,B), with a significant down-regulation in both the DIO and ob/ob groups. In the case of the BT, the multiple comparison post hoc test indicated that T1R3, T2R116, Gα14, αTransd and TRPM5 were downregulated in the obese DIO and ob/ob groups (Fig. 4C,D, Table 1).

The HP (Fig. 4E,F, Table 1) and Ctx (Fig. 4G,H, Supplemental Table S3) did not show the same extent of obesity-associated differences as observed in the HT and BT. Statistical analysis by ANOVA indicated that only HP showed effects of nutritional status (p = 0.001), but not the Ctx (p = 0.050). Only T2R116 was found to be significantly upregulated in the HP of DIO mice (Fig. 4E).

### Expression of sweet GPCTR subunits and bitter GPCTRs and associated a-subunits in the brain of fasted mice

We evaluated the effects of fasting on the GPCTR related gene expression by RT-PCR in HT, BT, HP and Ctx of lean C57BL/6 mice fed *ad libitum* and 16 hour fasted lean C57BL/6 mice. Statistical analysis by ANOVA indicated that fasting influenced GPCTR related gene expression only in BT and HP. No impact of 16 h fasting was observed in HT and Ctx. In the BT, T2R116, αTransd and TRPM5 showed a downregulation after fasting (Supplemental Table S4, Table 1). In the HP only Gα14 showed a downregulation after fasting (Supplemental Table S4, Table 1).

### Glucose and leptin modulate sweet taste receptors and Gα14 expression in hypothalamic neurons

**Figure 5:**
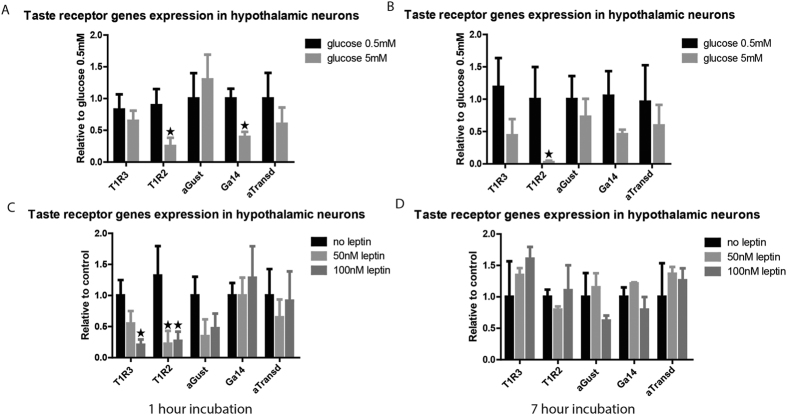
Sweet receptors and Gα14 in hypothalamic neurons are regulated by glucose and leptin levels. (**A**) Sweet taste receptor subunits and GPCTRs α subunit mRNA expression in a hypothalamic neuronal cell line after one hour incubation of high glucose levels. T1R2 and Gα14 were downregulated after incubation with high glucose. Glucose Treatment, p = 0.004; Gene, p = 0.587; Interaction, p = 0.220. (**B**) Sweet taste receptor subunits and GPCTRs α subunit mRNA expression in a hypothalamic neuronal cell line after 7 hour incubation of high glucose levels. T1R2 was downregulated after incubation with high glucose. Glucose treatment, p = 0.011; Gene, p = 0.494; Interaction, p = 0.158. (**C**) Sweet taste receptor subunits and GPCTRs α subunit mRNA expression in a hypothalamic neuronal cell line after one hour incubation high leptin concentrations. T1R2 and T1R3 were downregulated after incubation with high leptin. Leptin treatment, p = 0.019; Gene, p = 0.804; Interaction, p = 0.909. (**D**) Sweet taste receptor subunits and GPCTRs α subunit mRNA expression in a hypothalamic neuronal cell line after 7 hour incubation high leptin concentrations. No significant changes in expression following high leptin. Leptin treatment, p = 0.119; Gene, p = 0.548; Interaction, p = 0.931.

In order to investigate a possible mechanism for the nutritional regulation of GPCTR expression in the hypothalamus, we exposed an adult murine hypothalamic neuron derived cell line to different concentrations of glucose (0.5 mM (n = 8) and 5 mM (n = 8)) and leptin (0 nM (n = 12), 50 nM (n = 12) and 100 nM (n = 12)) for either 1 or 7 hours. We compared cells with additive to corresponding cells at the same time point and therefore used statistical analysis by two-way ANOVA. The analysis showed significant effects of glucose stimulation after 1 hour (p = 0.004) and after 7 hours (p = 0.011). Post-hoc analysis showed that higher glucose concentrations (5 mM), comparable to physiological levels during hyperglycemia, down-regulated specifically the sweet receptor subunit T1R2 after 1 (p = 0.038) and 7 hour (p = 0.014) as compared to the 0.5 mM glucose concentration (Fig. 5A,B). Higher glucose concentrations did not change the sweet receptor subunit T1R3 or the bitter receptor (T2R116, T2R11, T2R138) mRNA expression (data not shown). Only a trend for T1R3 down-regulation was observed after 7 hours incubation with high glucose (p = 0.062). Higher glucose concentrations rapidly down-regulated the expression of Gα14 after 1 hour (p = 0.049). Prolonged incubation (7 h) attenuated the down-regulation. Hyperglycemia did not influence the expression of αGust and αTransd (Fig. 5A,B).

Statistical analysis by two-way ANOVA showed significant effects of leptin stimulation after 1 hour (p = 0.019), but not after 7 hours (p = 0.119). A down-regulation in the expression of both sweet receptor subunits was observed. Multi-comparison post hoc test showed that during the 1 hour experiment T1R3 was down-regulated only after exposure to 100 nM leptin (p = 0.011), whereas T1R2 was down-regulated after exposure to both the 50 nM (p = 0.012) and 100 nM (p = 0.009) concentration (Fig. 5C,D). The bitter receptors (data not shown) and the GPCTRs downstream signalling pathway gene expression did not show any differences after either the 1 or 7 hour incubation (Fig. 5C,D).

### Expression of sweet GPCTR subunits and bitter GPCTR receptors and associated a-subunits in other oral and extra-oral organs

**Figure 6:**
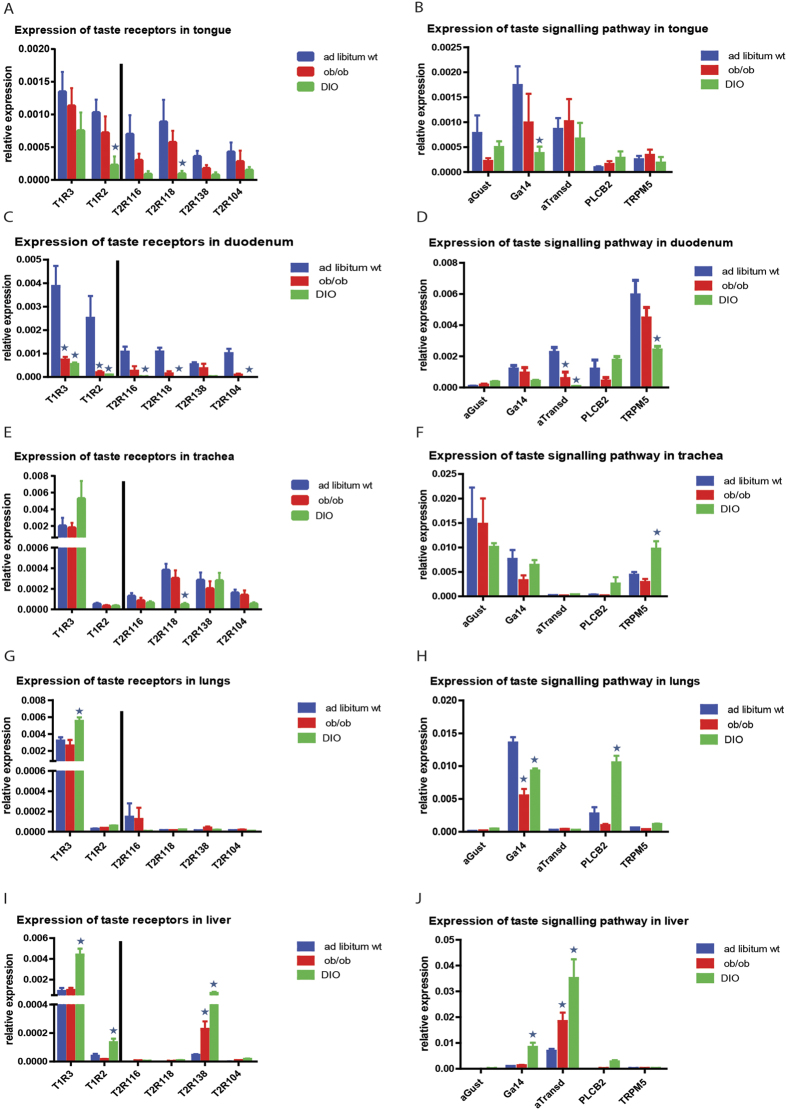
GPCTRs and downstream signaling mediator mRNA expression in oral and extra-oral organs is regulated by obesity. (**A**) Sweet and bitter taste receptor mRNA expression in tongue of *ad libitum* wild type and obese (ob/ob and DIO) mice. DIO animals showed reduced T1R2 and T2R118 expression. (**B**) GPCTR signalling pathway mRNA expression in tongue of *ad libitum* wild type and obese (ob/ob and DIO) mice. (**C**) Sweet and bitter taste receptor mRNA expression in duodenum of *ad libitum* wild type and obese (ob/ob and DIO) mice. T1R3 and T1R2 were downregulated in ob/ob and DIO animals. T2R116, T2R118, T2R104 showed a downregulation only in DIO animals. (**D**) GPCTR signalling pathway mRNA expression in duodenum of *ad libitum* wild type and obese (ob/ob and DIO) mice. αTransd was downregulated in obese (ob/ob and DIO) animals, TRPM5 was downregulated in DIO animals. (**E**) Sweet and bitter taste receptor mRNA expression in trachea of *ad libitum* wild type and obese (ob/ob and DIO) mice. T2R118 was decreased in DIO animals. (**F**) GPCTR signalling pathway mRNA expression in trachea of *ad libitum* wild type and obese (ob/ob and DIO) mice. (**G**) Sweet and bitter taste receptor mRNA expression in lungs of *ad libitum* wild type and obese (ob/ob and DIO) mice. T1R3 was upregulated in DIO mice. (**H**) GPCTR signalling pathway mRNA expression in lungs of *ad libitum* wild type and obese (ob/ob and DIO) mice. Gα14 was downregulated in obese (ob/ob and DIO) animals. PLCβ2 was upregulated in DIO animals. (**I**) Sweet and bitter taste receptor mRNA expression in liver of *ad libitum* wild type and obese (ob/ob and DIO) mice. T1R3 and T1R2 were upregulated in DIO animals, T2R138 was upregulated in ob/ob and DIO animals. J) GPCTR signalling pathway mRNA expression in liver of *ad libitum* wild type and obese (ob/ob and DIO) mice. Gα14 was upregulated in DIO mice, αTransd was upregulated in ob/ob and DIO mice.

Lean C57BL/6 mice were also analyzed on expression of sweet taste receptors subunits (T1R2 and T1R3) and bitter taste receptors (T2R116, T2R118, T2R138 and T2R104 throughout oral (tongue) and extra-oral tissues (trachea, lungs, liver, and duodenum) outside the central nervous system (Fig. 6). The tongue showed mRNA expression of all examined taste receptors, while the extra-oral tissues showed a more restricted expression pattern. T1R2 mRNA was particularly expressed in the duodenum and tongue. Similar, bitter receptors analyzed, like T2R116, showed high expression in tongue and duodenum (Fig. 6).

Of note, αGust and αTrans mRNA expression was found to be different among tissues: αGust mRNA being relatively high in the trachea and αTrans mRNA in the liver (Fig. 6). Gα14 mRNA expression was relatively high in lungs and trachea, followed by brain and tongue (Fig. 6).

### Obese mice show differential GPCTR expression as compared to lean mice in other oral and extra-oral organs

The above reported findings in lean mice were compared to those for obese animals (DIO and ob/ob mice). The tongue of DIO mice showed a prominent down-regulation of most GPCTR and related genes (Supplemental Figure S1b, Fig. 6A,B). Statistical analysis by ANOVA indicated significant differences of GPCTR mRNA expression in the tongue upon HFD feeding (p = 0.001). Multiple comparison post hoc testing indicated that T1R2 and T2R118 mRNA were significantly downregulated in DIO animals (Fig. 6A, Table 2). For the GPCTR signalling pathway expression, the post hoc testing indicated a downregulation of Gα14 in DIO mice (Fig. 6B, Table 2). The ob/ob group did not show differences with the *ad libitum* group in any of the analyzed GPCTR related genes.

For the duodenum (Supplemental Figure S1b, Fig. 6C,D, Table 2), statistical analysis by ANOVA also showed a clear effect of obesity (p = 0.001). T1R3 and T1R2 were down-regulated in DIO and ob/ob mice. T2R116, T2R118 and T2R104 expression were only down-regulated in DIO mice (Fig. 6C, Table 2). In the case of the GPCTR signalling pathway in the duodenum (Fig. 6D, Table 2), αTransd showed a down-regulation in the DIO and ob/ob groups, while TRPM5 only showed a down-regulation in the DIO group.

We also analyzed the trachea, lungs and liver. For the trachea (Supplemental Figure S1b, Fig. 6E,F, Table 2), statistical analysis by ANOVA indicated significant differences of GPCTR mRNA expression in the different groups (p = 0.001). Multiple comparison post hoc testing (Table 2) showed a down-regulation of T2R118 in the DIO group compared to the *ad libitum* group (Fig. 6E, Table 2). Within the trachea TRPM5 expression was upregulated in DIO mice (Fig. 6F, Table 2). Statistical analysis by ANOVA showed differences in expression between the different groups in the lungs as well (p = 0.010). Most of the taste receptors showed little or no mRNA expression, with exception of T1R3, which showed an upregulation in the DIO group (Fig. 6G, Supplemental Table S3). We could also detect GPCTR signalling pathway expression (Fig. 6H, Supplemental Table S3), mainly from Gα14, PLCβ2 and TRPM5. While Gα14 showed a down-regulation in the ob/ob group and the DIO group, PLCβ2 was upregulated in the DIO.

For liver, ANOVA analysis (Fig. 6I,J, Supplemental Table S3) showed again significant differences between lean versus obese animals (p = 0.001), however, we could only detect mRNA expression of the sweet receptor subunits T1R3 and T1R2 and the bitter receptor T2R138. The multiple comparisons post hoc testing indicated an upregulation of T1R3 and T1R2 in the DIO mice. T2R138 expression was higher in the obese DIO and ob/ob mice (Fig. 6I, Supplemental Table S3). For the GPCTRs signalling pathway expression in the liver (Fig. 6J, Supplemental Table S3), we could only detect mRNA expression of Gα14, αTransd and PLCβ2. Gα14 was upregulated in the DIO mice and αTransd was upregulated in both DIO and ob/ob animals.

### Expression of sweet GPCTR subunits and bitter GPCTRs and associated a-subunits in other oral and extra-oral organs of fasted mice

We also studied the impact of 16h fasting on GPCTR related gene expression in oral and extra-oral tissues. The GPCTR related gene expression was affected in the tongue, trachea and duodenum after fasting. Multiple comparisons post hoc testing (Supplemental Table S5, Table 2) indicated that in the tongue T1R2, T2R118 and Gα14 were significantly down-regulated after fasting. In the duodenum, multiple comparison post hoc analysis showed that only the bitter receptors T2R116, T2R118 and T2R104 were significantly down-regulated in the fasting group (Supplemental Table S5, Table 2). In the GPCTR signalling pathway in the duodenum (Supplemental Table S5, Table 2) TRPM5 showed an upregulation in the fasting group.

In trachea, multiple comparisons post hoc testing (Supplemental Table S5, Table 2) showed that T1R3, αGust and TRPM5 were significantly upregulated in the fasting state. As in the trachea, also in the lungs T1R3 was found upregulated after fasting (Supplemental Table S5, Supplemental Table S3).

## Discussion

Sweet taste motivates the ingestion of potentially caloric or rewarding components of food, while bitter taste induces aversive behaviors to avoid potentially harmful components in the food^5^. Intriguingly, sweet and bitter taste receptors are also expressed in extra-oral tissues, including the central nervous system^21^. While the role of sweet and bitter GPCTRs in oral tissue in eating behavior has been widely studied, information on the presence and possible role of taste receptors in extra-oral tissues is relatively scarce^15^, let alone if they are known to modulated by dietary inputs. The present study shows that sweet and bitter GPCTRs are present in different brain areas such as hypothalamus, hippocampus, brainstem and cortex and that different metabolic states, like obesity and fasting, can modulate their expression specifically in the hypothalamus and brainstem, areas key for metabolic regulation. Furthermore we characterize for the first time the expression of GPCTRs and their signalling molecules in select extra-oral tissues and demonstrate their modulation under various nutrient stresses.

Our investigation started with exploring the presence of sweet (T1R3 and T1R2) and bitter receptors (T2R116, T2R118, T2R138 and T2R104) in the brain. Expression of genes for the sweet receptor subunits T1R3 and T1R2 and bitter receptors T2R104 and T2R138 in the hypothalamus and brainstem has been reported before^23^,^24^. Using quantitative PCR and *in situ* hybridization our study recapitulates the earlier findings that sweet GPCTRs T1R3 and T1R2 mRNAs are more prominent in the hypothalamus and brainstem as compared to the hippocampus and cortex. We moreover noted that bitter GPCTRs genes are comparatively less expressed in brain, with the exception of the T2R116 gene. This receptor, of which the human orthologue recognizes β-glucosylated tastants^4^, shows a similar expression pattern as the sweet GPCTRs, with higher expression in the hypothalamus and brainstem. Closer analysis of the anatomical distribution of GPCTR gene expression in the hypothalamus and brainstem by *in situ* hybridization revealed that T2R116 is mainly expressed in ARC, VMH and the dorsal DMH of the hypothalamus and caudal NTS of the brainstem. Expression of T1R2 and T1R3 genes is relatively high in the ARC of the hypothalamus, as also reported previously^24^. Of note, the ARC, VMH, DMH in the hypothalamus and the NTS in the brainstem are well known components of the brain circuits involved in sensing of nutrients and regulation of energy metabolism^30^. Taste receptors are coupled to a heterotrimeric G protein consisting of an α-, β- and y-subunits. αGust, the α-subunit usually coupled to the taste receptor G protein, was poorly expressed in brain regions with high GPCTR expression. Mice lacking αGust expression preserve behavioral and taste afferent nerve responses to higher concentrations of several bitter and sweet compounds^7^. The proteins αTransd and Gα14 are known to act as alternative α-subunits coupled to GPCTRs in the tongue^31^. Gα14 and αGust are expressed in a mutually exclusive fashion in different subsets of taste cells in the tongue^9^,^11^,^31^. Gα14 expression was previously detected in brain, however the specific anatomical distribution was not examined^31^. Our investigation revealed a clear expression of Gα14 and some αTransd mRNA in the brainstem. More detailed anatomical analysis showed that Gα14 is mainly expressed in energy homeostasis regulating areas of the hypothalamus and brainstem, such as the ARC, VMH, DMH and NTS. Co-localization experiments showed that some of the ARC and NTS T2R116-positive cells also express Gα14 and PLCβ2. It is conceivable that GPCTRs in the hypothalamus and brainstem also employ Gα14 as α-subunit, as described for some taste cells of the tongue^9^,^11^,^31^. This possibility warrants further research.

The presence of GPCTRs and associated signalling components in brain areas known to be involved in energy homeostasis opens the possibility that the brain not only integrates taste information arriving via afferent inputs from taste cells located in extra-oral organs, but also directly senses tastants and responds to such signals. Stimulated by these observations, we next compared GPCTR expression in brains of lean and obese animals, either being overweight due to leptin deficiency (ob/ob mice) or to high fat diet feeding (DIO mice). We observed a down-regulation of GPCTR expression in the brains of both types of obese mice. This down-regulation was specifically observed in the hypothalamus and brainstem, and not in the hippocampus and cortex. These results confirmed earlier reported down-regulation of sweet GPCTR expression in the hypothalamus of ob/ob mice^24^, and extended this to DIO mice. It is known that disruptions of the hypothalamic-brainstem nutrient sensing circuit can promote the development of obesity and diabetes^32^,^33^. Experiments with cultured hypothalamic neural cells revealed that exposure to high levels of glucose or leptin down-regulates expression of the sweet receptor subunit T1R2. Only leptin administration was found to result in significant reduction of T1R3 expression too. Tasting of glucose through T1R heterodimers, and possibly even monomers, is thought to contribute to regulation of glucose homeostasis^34^. The observed differential response of components of the T1R family to high glucose is in agreement with previous reports^24^,^35^. Analysis of bitter receptor expression gave a different picture. Only expression of the bitter receptor T2R116 was found to be significantly lower in brains of obese mice. Glucose and leptin did not modify expression of any of the examined bitter receptor genes in cultured hypothalamic neurons. Previously, an intriguing hypothesis for the functional role of T2R16 was proposed^36^. In this study, evidence for a positive evolutionary selection of T2R16 was presented, followed by the suggestion that an improved sensitivity to β-glucopyranosides in humans that present the positive selected allele N172, might be translated in an increased protection against β-glucopyranosides, like cyanogenic compounds found in natural toxins. The decreased expression observed in oral and extra-oral tissues might indicate a decrease of sensitivity to this compounds during obesity.

Finally we looked into the expression of GCPTRs and their associated signalling components in other tissues such as tongue, duodenum, lung, liver and trachea. While the expression of T1R3 was generally the highest among sweet receptors in all these tissues, T1R2 was mainly expressed in the tongue and duodenum. It is known that the T1R3+T1R2 heterodimer mediates sweet responses, while the T1R1+T1R3 heterodimer mediates umami responses^1^,^2^. The differential expression of T1R3 and T1R2 indicates that T1R3 expression might also be related to umami sensing in oral and extra-oral tissues^37^. Similar to the distribution observed for T1R2, all analysed bitter receptors mRNAs were particularly detected in the tongue and duodenum. The required cascade components for signaling (a-subunits, PLCb2 and TRPM5) were all detectable in tongue, trachea and duodenum.

αGust expression was most prominent in trachea and tongue; lungs and duodenum showed a more modest expression. Expression of αGust has been demonstrated before in taste cells in the upper airways^38^. It was stipulated that these taste cells might contribute to cough, bronchodilation or bronchoconstriction responses in order to protect the airways from harmful inhalants^17^,^18^. The expression of αGust in the duodenum seems restricted to entero-endocrine cells, where it mediates glucagon like peptide-1 and ghrelin secretion and thus gastric emptying after food ingestion^25^,^39^. We observed expression of αTransd mainly in liver, tongue and duodenum. The presence of αTransd in taste cells in the tongue and GI tract has also previously been demonstrated^22^,^40^. The physiological importance of αTransd as a relay of taste information was illustrated by the partial rescue of behavioral and taste afferent nerve responses to sweet and bitter compounds after introducing a transgene in which αTransd is expressed under the control of the αGust promoter in αGust KO mice^12^. Surprisingly, we noted a high expression of αTransd in the liver with a concomitant very limited expression of other GPCTR signaling pathway genes. The demonstration of a variety of GPCTRs in tongue, duodenum and liver supports the idea that these organs too can act as sensors for different nutrient signals and contribute directly to the regulation of food intake^20^,^41^.

The noted presence of GPCTRs and components of associated signalling pathways in extra-oral tissues, particularly the brain, suggests that these tissues may be involved in chemo-sensing with potential implications for food intake and energy homeostasis, as well. Several studies have indeed provided evidence that there is a relation between metabolic state and taste responses. Different metabolic states not only influence motivation and food intake behaviour, but also modulate the response to gustatory stimuli at the level of the taste bud, afferent taste signals or integrative brainstem sites, like the NTS and the parabrachial nucleus^42^,^43^. Our analysis of GPCTR expression during feast (obesity) and famine (fasting) showed that especially obesity, but also fasting, modify the expression of sweet and bitter GPCTRs in both oral and extra-oral tissues. Of interest in this respect is that GPCTR pathway components are not only differentially expressed in the brains of obese mice as compared to lean counterparts, but also in other tissues. For example, we observed a decreased sweet and bitter GPCTR expression in the hypothalamus and brainstem, but also in the tongue and duodenum of obese animals. It has previously been shown that obesity influences the sensing of sweet and bitter tastants in humans and rodents^28^,^42^,^44^. It has been suggested that sweet and bitter taste sensitivity could be related to the susceptibility of developing obesity and diabetes. This idea is based on results from different correlational studies with obese and diabetic patients^28^,^45^,^46^,^47^, as well as data from taste receptor or taste receptor signalling pathway KO mice^48^,^49^. However, the mechanism for the relationship between a decrease taste sensitivity and the onset of metabolic disorders is still far from clear. In a broader perspective it seems surprising that a down-regulation of sweet and bitter taste receptors would promote obesity, instead of having the opposite effect. However, a down-regulation in sweet taste receptors might promote obesity by increasing food intake in order to compensate for the reduced sensitivity, whereas a down-regulation of bitter taste receptors promotes obesity by allowing the use of additional food sources.

Our observations with obese animals are in accordance with an involvement of taste receptors in these metabolic disorders. For instance we found Gα14 to be reduced during obesity in the tongue and previously it has been proposed, based on bioinformatic analysis, that Gα14 is directly linked to obesity and obesity related pathologies^50^. In the current study TRPM5 was found to be modulated by obesity in trachea and duodenum, as well as in the brainstem. Others showed that TRPM5 is required for sweet, bitter and umami taste responses^1^; and its expression and functionality have been related to the development of diabetes by modulating insulin secretion and glucose homeostasis^49^,^51^. It is likely that the decreased expression of GPCTRs during obesity will result in a different threshold for glucose or amino acids in brain areas highly sensitive to hormone and metabolite changes in plasma, like the hypothalamus and brainstem. Specific desensitization for β-glucopyranosides, i.e., T2R16 ligands, was previously reported after repeated *in vitro* and *in vivo* stimulations in humans^52^.

Some of the differences we observed between obese DIO and ob/ob mice are of particular interest. Ob/ob animals do not show the prominently reduced sweet and bitter GPCTR expression in the tongue, like the DIO animals. Different studies have demonstrated that leptin can diminish the preference for sweet substances, whereas mice deficient in the leptin receptor Ob-Rb (db/db) do not show a reduced sweet taste sensitivity^53^,^54^. Similarly, we found bitter GPCTR expression to be reduced in the tongue and duodenum of DIO mice, but less in ob/ob mice. This may point, but does not proof, a role for leptin. Of interest, the expression of some bitter GPCTRs in the GI was reported to be reduced when mice were fed a high cholesterol diet^55^. Furthermore it was proposed that cholesterol can modulate T2R family member transcription by SREBP-2 in the proximal intestine^56^. In view of the proposed regulatory effect of SREBP-2, the noted upregulated expression of bitter GPCTRs in liver of obese animals is remarkable and warrants further investigation.

We finally also studied the impact of fasting on the expression of different components of the GPCRT pathway in the various tissues. A fasting period of 16 hours did not result in any prominent or consistent changes at the mRNA level, suggesting that acute metabolic changes, such as those induced by fasting, may have a lesser impact on the GPCRT expression than more chronic metabolic changes. However, further insight at the level of post-translational modification is necessary before such a conclusion can be reached with more certainty.

In conclusion, our study confirms that sweet and bitter GPCTRs and their signalling pathways are expressed not only in oral but also extra-oral tissues. Moreover, we provide evidence that in the central nervous system brain regions involved in the regulation of energy homeostasis, such as hypothalamus and brainstem, also express the necessary components of sweet and bitter GPCTR signalling. Additionally, we show that their expression differs between lean and obese mice, both in brain and peripheral tissues. Although the specific impact that this modulation of GPCTR signalling has on the development of obesity or diabetic disorders still has to be elucidated, our study shows that a direct role of the brain in tastant sensing and subsequent regulation of energy homeostasis should not be excluded and deserves further research.

## Additional Information

**How to cite this article**: Herrera Moro Chao, D. *et al*. Impact of obesity on taste receptor expression in extra-oral tissues: emphasis on hypothalamus and brainstem. *Sci. Rep.* **6**, 29094; doi: 10.1038/srep29094 (2016).

## Supplementary Material

Supplementary Information

## Figures and Tables

**Table 1:** Effects of obesity and fasting on GPCTRs expression in brain areas.

*Sweet receptors subunits*	HYPOTHALAMUS			BRAINSTEM			HIPPOCAMPUS		
	ob/ob	DIO	Lean Fasting	ob/ob	DIO	Lean Fasting	ob/ob	DIO	Lean Fasting
T1R3	0.009	0.010	0.575	<0.001	<0.001	0.418	0.402	0.265	0.957
T1R2	0.949	0.360	0.518	0.889	0.871	0.877	0.998	0.474	0.938
*Bitter receptors*	ob/ob	DIO	Lean Fasting	ob/ob	DIO	Lean Fasting	ob/ob	DIO	Lean Fasting
T2R116	0.757	0.909	0.833	0.013	0.026	0.018	0.821	0.016	0.783
T2R118	0.971	0.954	0.972	0.972	0.974	0.980	0.986	0.995	1.00
T2R138	0.855	0.982	0.967	0.899	0.880	0.878	0.759	1.00	0.964
T2R104	0.960	0.988	0.986	0.998	0.995	0.994	0.972	0.985	0.978
*GPCTRs signalling pathways*	ob/ob	DIO	Lean Fasting	ob/ob	DIO	Lean Fasting	ob/ob	DIO	Lean Fasting
Gα14	0.045	0.020	0.248	<0.001	<0.001	0.998	0.130	0.634	0.015
αGust	0.988	0.933	0.992	0.972	0.977	0.975	0.999	0.695	0.999
αTransd	0.920	0.952	0.951	0.044	0.049	0.040	0.731	0.929	0.963
PLCβ2	0.953	0.797	0.812	0.753	0.564	0.733	0.184	0.760	0.557
TRPM5	0.995	0.783	0.788	<0.001	<0.001	<0.001	0.881	0.462	0.474

P-value after pairwise comparison LSD *post hoc* test in the different nutritional status groups compared to the *ad libitum* control group. P-values < 0.05 are depicted in bold.

**Table 2:** Effects of obesity and fasting on GPCTRs expression in tongue, trachea and duodenum.

*Sweet receptors subunits*	TONGUE			TRACHEA			DUODENUM		
	ob/ob	DIO	Lean Fasting	ob/ob	DIO	Lean Fasting	ob/ob	DIO	Lean Fasting
T1R3	0.936	0.194	0.255	0.934	0.495	0.006	<0.001	<0.001	0.791
T1R2	0.693	0.013	0.007	1.00	0.996	0.996	<0.001	<0.001	0.512
*Bitter receptors*	ob/ob	DIO	Lean Fasting	ob/ob	DIO	Lean Fasting	ob/ob	DIO	Lean Fasting
T2R116	0.245	0.057	0.070	0.977	0.972	0.987	0.055	0.049	0.045
T2R118	0.466	0.009	0.008	0.992	0.036	0.807	0.051	0.041	0.047
T2R138	0.570	0.289	0.288	0.986	0.972	0.966	0.549	0.370	0.375
T2R104	0.307	0.267	0.227	0.998	0.536	0.989	0.051	0.047	0.049
*GPCTRs signalling pathways*	ob/ob	DIO	Lean Fasting	ob/ob	DIO	Lean Fasting	ob/ob	DIO	Lean Fasting
Gα14	0.238	<0.001	<0.001	0.057	0.620	0.828	0.636	0.116	0.379
αGust	0.113	0.579	0.702	0.775	0.500	<0.001	0.803	0.576	0.790
αTransd	0.729	0.137	0.117	0.988	0.949	0.986	0.001	<0.001	0.731
PLCβ2	0.872	0.420	0.966	0.958	0.388	0.962	0.085	0.547	0.658
TRPM5	0.928	0.942	0.315	0.617	0.227	0.037	0.118	<0.001	<0.001

P-value after pairwise comparison LSD *post hoc* test in the different nutritional status groups compared to the *ad libitum* control group. P-values < 0.05 are depicted in bold.
