# Supplementary material for: Impact of obesity on taste receptor expression in extra-oral tissues: emphasis on hypothalamus and brainstem
Source: Sci Rep. 2016 Jul 8;6:29094. doi: 10.1038/srep29094 (PMC4937374; doi:10.1038/srep29094)
Supplement: Supplementary Information [file srep29094-s1.pdf]

Impact of obesity on taste receptor expression in extra-oral tissues: emphasis on hypothalamus and brainstem

Herrera Moro Chao D[1, 2], Argmann C[5], Van Eijk M[1,3], Boot RG[3], Ottenhoff R[1], Van Roomen C[1], Foppen E[2], Siljee JE[2], Unmehopa UA[2], Kalsbeek A[2,4], Aerts JMFG[1,3]*

[1]Department of Medical Biochemistry, Academic Medical Center, Amsterdam, 1105 AZ, The Netherlands.

[2]Department of Endocrinology and Metabolism, Academic Medical Center, Amsterdam, 1105 AZ, The Netherlands.

[3]Department of Biochemistry, Leiden Institute of Chemistry, Leiden, 2333 CC, The Netherlands.

[4]Hypothalamic Integration Mechanisms, Netherlands Institute for Neuroscience, Amsterdam, 1105 BA, The Netherlands.

[5]Department of Genetics and Genomic Sciences, Icahn Institute for Genomics and Multiscale Biology, Icahn School of Medicine at Mount Sinai, New York, NY 10029, USA.

***Corresponding author**

E-mail: j.m.f.g.aerts@lic.leidenuniv.nl (JMFGA)

Supplemental Information

Methods

Dissection of brain areas

The BT was isolated from the rest of the brain by separating the hindbrain from the cortical lobes at the level of inferior colliculus. The pons and cerebellum were separated from the BT and the BT was cut slightly lower of the obex to separate it from the spinal cord. For the HP a small curved blunt forceps was introduced between the cerebral hemispheres in a closed position, slowly opening the forceps the cortex was separated from the HP. For the Ctx, slices of 300 µm from the enlargement of the lateral ventricle and beginning of the putamen were made. Using a steel needle, the Ctx was dissected. Before HT dissection, the frontal part of the brain was cut coronally to separate the forebrain areas. After that, blunt curved forceps were introduced at the level of the mammillary nucleus and the HT was separated from the rest of the brain.

LNA In situ Hybridization probes

LNA-20 -O-methyl-RNA probes specific for the T2R116 and Gα14 mouse mRNA sequence were FAM tagged at the 50 end and custom ordered (Ribotask, Langeskov, Denmark). The antisense probe for T2R116 was the following: 5’- lA mA mG lC mC mU lT mU mC lT mC mA lT mC mU lT mA mG lC 3’, while the sense probe T2R116 was: 5’ lG mC mU lA mA mG lA mU mG lA mG mA lA mA mG lG mC mU lT 3’. The antisense probe for Gα14 was the following: 3’ IT mC mU IT mU mC IT mC mA IG mA mG IT mC mG IC mG mU IA 5’, while the sense probe Gα14 was: 5’ IA mG mA IA mA mG IA mG mU IC mU mC IA mG mC IG mC mA IT 3’. In all the probes the ‘m’ symbolizes 20 -O-methylRNA (mA, mG, mC, and mU) and ‘l’ symbolizes LNA bases.

Figure legends

Supplemental figure S1. A) One directional axis heat map of mRNA expression of sweet and bitter receptors and its signalling pathways in hypothalamus, brainstem, hippocampus and cingulate cortex. Obesity (ob/ob and DIO) and fasting modify the GPCTRs expression in the brain. B) One directional axis heat map of mRNA expression of sweet and bitter receptors and its signalling pathways in oral and extra-oral organs.

A

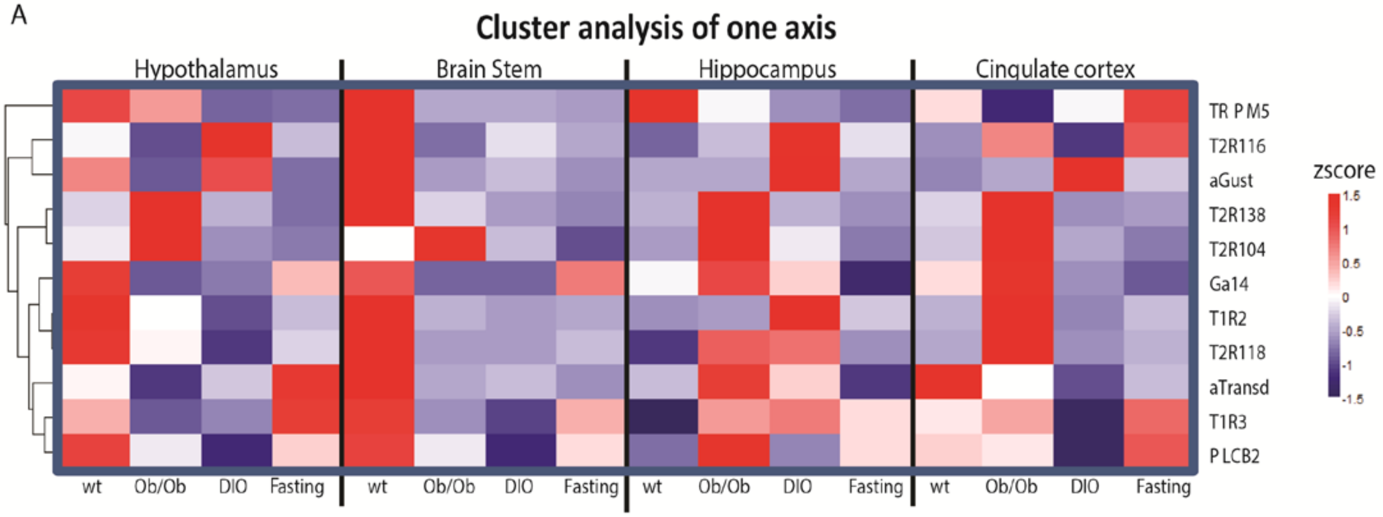
Cluster analysis of one axis
Hypothalamus
Brain Stem
Hippocampus
Cingulate cortex
TR P M5
T2R116
aGust
T2R138
T2R104
Ga14
T1R2
T2R118
aTransd
T1R3
P LCB2
wt
Ob/Ob
DIO
Fasting
zscore
1.5
1
0.5
0
-0.5
-1
-1.5

B

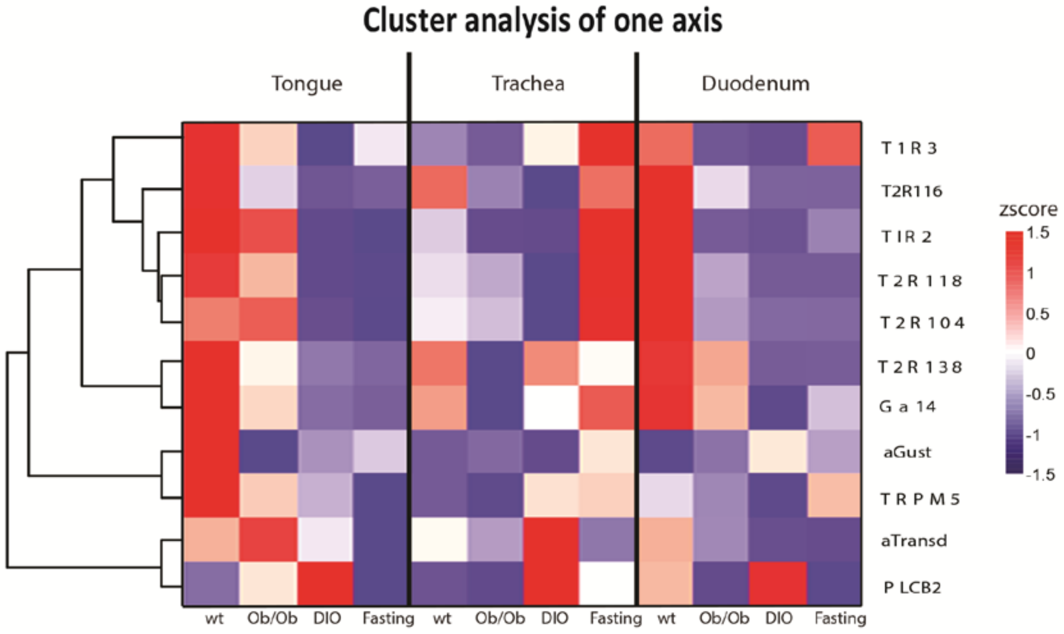
Cluster analysis of one axis
Tongue
Trachea
Duodenum
T 1 R 3
T2R116
T IR 2
T 2 R 1 1 8
T 2 R 1 0 4
T 2 R 1 3 8
G a 14
aGust
T R P M 5
aTransd
P LCB2
wt
Ob/Ob
DIO
Fasting
zscore
1.5
1
0.5
0
-0.5
-1
-1.5

Supplemental Table S1. Metabolic parameters in obese and lean animals. Body weight, OGTT, HOMA-IR and plasma hormone and metabolite concentrations in obese (DIO and ob/ob) and lean mice. ND=not detectable. NA=not assessed. * indicates significant differences compared to the *ad libitum* controls.

Metabolic parameter	Lean	DIO	Ob/ob
**Body weight**	25.4±0.6	41.2±2*	45.3±1.3*
**Insulin (ng/ml)**	1.1±0.2	2.9±0.1*	25.4±6.90*
**Leptin (ng/ml)**	3.1±0.1	70.2±14.6*	ND
**HOMA-IR**	5.8±0.9	31.4±11.2*	370±222*
**OGTT**	821±186.6	1899.8±91.4*	NA
**Glucose (mM)**	5.2±0.3	10.1±0.5*	11.4±3.3*
**Daily food intake**	2.6±0.1	3.1±0.2	5.5±0.6*

Supplemental Table S2. Primers used for RT PCR of mRNA of taste receptors and GPCTRs signalling pathways expressed in oral and extra-oral organs.

**Sweet and bitter receptors**	
T1R3	FWD: 5'- aggtggctcacagttctgct -3'<br>REV: 5'- gaggtgagccattggttgtt -3'
T1R2	FWD: 5'- catggtgcaactgatggttc -3'<br>REV: 5'- ctctcggccataatcgtcat -3'
T2R116	FWD: 5'- atgcagagatgccagcacta -3'<br>REV: 5'- gaagcagagcactccaaacc -3'
T2R118	FWD: 5'- caacgcaagtcaccatcttc -3'<br>REV: 5'- ggctgatgagaatcgtctcc -3'
T2R138	FWD: 5'- tatctccctggggagtcaca -3'<br>REV: 5'- agcagcacagaatgacacca -3'
T2R104	FWD: 5'- tccctgttgccattccttta -3'<br>REV: 5'- tctgcagtgccctcatagtg -3'
**GPCTRs signalling pathways**	
Gα14	FWD: 5'- ctctggtggcaatctccaat a-3'

	REV: 5'- catcatcgttggtgttcagg -3'
αGust	FWD: 5'- tctacattcccgggtgaaaa -3'<br><br>REV: 5'- gcaggtgactccttcaaagc -3'
αTransd	FWD: 5'- tgcctatgacatggtgcttg -3'<br><br>REV: 5'- atggaggtggttgcgaagta -3'
PLCβ2	FWD: 5'- ttccctttcctggacacttg -3'<br><br>REV: 5'- cccaagatgcagcctagaag -3'
TRPM5	FWD: 5'- gggctgagagagcaagaaaa -3'<br><br>REV: 5'- ggagccagtgtatccgtcat -3'

Supplemental Table S3. Effects of obesity and fasting on GPCTRs expression in lungs, liver and Ctx. P-value after pairwise comparison LSD *post hoc* test in the different nutritional status groups compared to the *ad libitum* group. P-values <0.05 are depicted in bold. NE = not expressed.

	LUNGS			LIVER			CINGULAR CORTEX		
Sweet receptors subunits	**ob/ob**	**DIO**	**Lean Fasting**	**ob/ob**	**DIO**	**Lean Fasting**	**ob/ob**	**DIO**	**Lean Fasting**
**T1R3**	0.833	**0.046**	0.998	0.833	**0.043**	0.999	0.995	0.891	0.978
**T1R2**	0.994	0.885	0.952	0.569	**0.042**	0.602	0.947	0.992	0.979
Bitter receptors	**ob/ob**	**DIO**	**Lean Fasting**	**ob/ob**	**DIO**	**Lean Fasting**	**ob/ob**	**DIO**	**Lean Fasting**
**T2R116**	0.995	0.762	0.765	NE	NE	NE	0.922	0.919	0.964
**T2R118**	0.972	0.958	0.996	NE	NE	NE	0.973	0.979	0.995
**T2R138**	0.965	0.974	0.995	**0.035**	**<0.001**	0.702	0.705	0.905	0.927
**T2R104**	0.995	0.988	0.996	NE	NE	NE	0.971	0.981	0.973
GPCTRs signalling pathways	**ob/ob**	**DIO**	**Lean Fasting**	**ob/ob**	**DIO**	**Lean Fasting**	**ob/ob**	**DIO**	**Lean Fasting**
**Gα14**	**<0.001**	**0.001**	0.558	0.958	**0.036**	0.875	0.629	0.384	0.067
**αGust**	0.980	0.778	0.952	NE	NE	NE	1.00	0.983	0.996
**αTransd**	0.953	0.950	0.941	**<0.001**	**<0.001**	0.916	0.890	0.616	0.889
**PLCβ2**	0.076	**<0.001**	0.550	0.988	0.593	0.319	0.953	0.564	0.773
**TRPM5**	0.905	0.623	0.945	NE	NE	NE	0.940	0.983	0.930

Supplemental Table S4. Effects of 16 hour fasting on GPCTRs expression in brain areas. Taste receptor and taste signalling pathway components relative expression in brain areas of *ad libitum* wild type and 16 hour fasted mice. P-values <0.05 are depicted in bold.

	**HYPOTHALAMUS**		**BRAINSTEM**		**HIPPOCAMPUS**		**CINGULAR CORTEX**	
Sweet receptors subunits	***Ad libitum wt***	**Lean Fasting**	***Ad libitum wt***	**Lean Fasting**	***Ad libitum wt***	**Lean Fasting**	***Ad libitum wt***	**Lean Fasting**
**T1R3**	0.007± 0.001	0.009± 0.0024	0.009± 0.002	0.006± 0.0007	0.002± 0.001	0.004± 0.0007	0.002± 0.0003	0.003± 0.0003
**T1R2**	0.0001± 0.00007	0.00006± 0.00001	0.0001± 0.00003	0.00001± 0.000002	0.00002± 0.000006	0.00002± 0.00007	0.00001± 0.000004	0.00002± 0.000002
Bitter receptors	***Ad libitum wt***	**Lean Fasting**	***Ad libitum wt***	**Lean Fasting**	***Ad libitum wt***	**Lean Fasting**	***Ad libitum wt***	**Lean Fasting**
**T2R116**	0.0003± 0.0001	0.0002± 0.0001	**0.0005± 0.0002**	**0.0001± 0.00001**	0.00007± 0.00003	0.00005± 0.00004	0.00002± 0.000007	0.00008± 0.00005
**T2R118**	0.00003± 0.00002	0.00001± 0.000006	0.00004± 0.00003	0.000007± 0.000001	0.00001± 0.000004	0.00002± 0.00001	0.000009± 0.000003	0.00001± 0.000003
**T2R138**	0.00004± 0.00001	0.00001± 0.000005	0.00008± 0.00002	0.000004± 0.0000007	0.00002± 0.000004	0.00003± 0.00001	0.00002± 0.000009	0.000009± 0.000002
**T2R104**	0.00001± 0.000004	0.00002± 0.000001	0.000002± 0.000001	0.0000006± 0.0000002	0.000003± 0.000001	0.000002± 0.000005	0.000008± 0.000004	0.000003± 0.000002
GPCTRs signalling pathways	***Ad libitum wt***	**Lean Fasting**	***Ad libitum wt***	**Lean Fasting**	***Ad libitum wt***	**Lean Fasting**	***Ad libitum wt***	**Lean Fasting**
**Gα14**	0.004± 0.0009	0.003± 0.0005	0.003± 0.0006	0.003± 0.0001	**0.001± 0.0004**	**0.0004± 0.00003**	0.001± 0.0003	0.001± 0.0001
**αGust**	0.00001± 0.000006	0.00001± 0.000001	0.00001± 0.000009	0.0000003± 0.0000001	0.0000009± 0.0000008	0.0000001± 0.0000001	0.0000009± 0.0000006	0.0000009± 0.0000007
**αTransd**	0.0001± 0.00002	0.0002± 0.00008	**0.0007± 0.0002**	**0.00004± 0.000007**	0.00007± 0.00002	0.00001± 0.000003	0.0002± 0.00004	0.0001± 0.00004
**PLCβ2**	0.0002± 0.00008	0.0002± 0.0001	0.0002± 0.00006	0.0001± 0.00002	0.0001± 0.00006	0.0003± 0.00005	0.0002± 0.00003	0.0002± 0.00009
**TRPM5**	0.002± 0.00007	0.00009± 0.00003	**0.004± 0.001**	**0.0001± 0.00003**	0.0008± 0.0003	0.0002± 0.00006	0.00004± 0.000005	0.00006± 0.00002

Supplemental Table S5. Effects of 16 hour fasting on GPCTRs expression in oral and extra-oral organs. Taste receptor and taste signalling pathway components relative expression in oral and extra-oral organs of *ad libitum* wild type and 16 hour fasted mice. P-values <0.05 are depicted in bold. NE = not expressed.

	TONGUE		TRACHEA		DUODENUM		LIVER	
Sweet receptors subunits	***Ad libitum wt***	**Lean Fasting**	***Ad libitum wt***	**Lean Fasting**	***Ad libitum wt***	**Lean Fasting**	***Ad libitum wt***	**Lean Fasting**
**T1R3**	0.001± 0.0003	0.001± 0.0001	**0.002± 0.0004**	**0.01± 0.002**	0.003± 0.0008	0.004± 0.001	0.0008± 0.0003	0.0006± 0.0001
**T1R2**	**0.001± 0.0004**	**0.0001± 0.00003**	0.00005± 0.00001	0.0001± 0.00005	0.002± 0.0009	0.001± 0.000001	0.00003± 0.00001	0.00001± 0.000007
Bitter receptors	***Ad libitum wt***	**Lean Fasting**	***Ad libitum wt***	**Lean Fasting**	***Ad libitum wt***	**Lean Fasting**	***Ad libitum wt***	**Lean Fasting**
**T2R116**	0.0006± 0.0002	0.0001± 0.00004	0.0001± 0.00002	0.0001± 0.00003	**0.001± 0.0002**	**0.00001± 0.000001**	NE	NE
**T2R118**	**0.0008± 0.0003**	**0.00005± 0.00002**	0.0003± 0.00006	0.0009± 0.0004	**0.001± 0.0001**	**0.000001± 0.0000005**	NE	NE
**T2R138**	0.0003± 0.00008	0.00006± 0.00002	0.0002± 0.00007	0.0002± 0.00007	0.0005± 0.0001	0.00001± 0.000004	0.00004± 0.000008	0.0000008± 0.0000001
**T2R104**	0.0004± 0.0001	0.0001± 0.00003	0.0001± 0.00003	0.0003± 0.0001	**0.0009± 0.0002**	**0.0000003± 0.0000002**	NE	NE
GPCTRs signalling pathways	***Ad libitum wt***	**Lean Fasting**	***Ad libitum wt***	**Lean Fasting**	***Ad libitum wt***	**Lean Fasting**	***Ad libitum wt***	**Lean Fasting**
**Gα14**	**0.001± 0.0006**	**0.0003± 0.0001**	0.007± 0.001	0.008± 0.001	0.001± 0.0002	0.0006± 0.00008	0.0001± 0.00007	0.0008± 0.001
**αGust**	0.0007± 0.0003	0.0005± 0.0001	**0.01± 0.006**	**0.03± 0.001**	0.00006± 0.00001	0.00006± 0.00001	NE	NE
**αTransd**	0.0008± 0.0002	0.0004± 0.0002	0.0001± 0.00002	0.00008± 0.00003	0.002± 0.0002	0.001± 0.0009	0.006± 0.0007	0.003± 0.001
**PLCβ2**	0.0002± 0.00008	0.0002± 0.0001	0.0002± 0.0001	0.0009± 0.0004	0.001± 0.0005	0.0008± 0.0007	0,000004± 0,000003	0,000002± 0,0000002
**TRPM5**	0.00008± 0.00002	0.00003± 0.00002	**0.004± 0.0006**	**0.010± 0.003**	**0.005± 0.001**	**0.009± 0.0009**	NE	NE
